# Supplementary material for: The missing Northern European winter cooling response to Arctic sea ice loss
Source: Nat Commun. 2017 Mar 6;8:14603. doi: 10.1038/ncomms14603 (PMC5343504; doi:10.1038/ncomms14603)
Supplement: Supplementary Information — Supplementary Figures [file ncomms14603-s1.pdf]

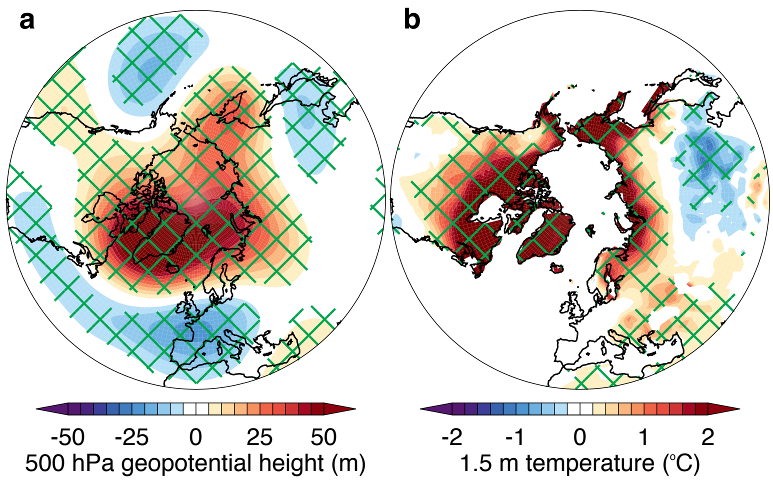

Supplementary Figure 1 | Climatological response to Arctic sea ice loss. a, Midwinter (January-February) 500 hPa geopotential height differenced between the low ice (LI) and high ice (HI) simulations (LI minus HI). Green hatching denotes differences that are statistically significant at the 95% (p = 0.05) confidence level. **b**, As **a** but for 1.5 m temperature.

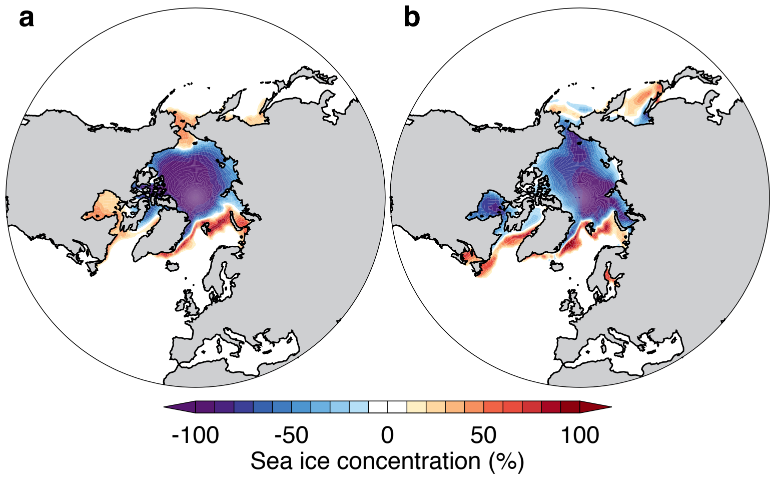

Supplementary Figure 2 | Differences in Arctic sea ice loss prescribed in the model simulations. a, Differences in early winter (November-December) sea ice concentration change between the low ice (LI) and high ice (HI) simulations compared to that between the 21st century (C21) and 20th century (C20) simulations ([C21-C20] minus [LI-HI]). Blue shading shows regions of larger losses of ice between C21 and C20 than between LI and HI. Red shading shows regions of larger losses of ice between LI and HI than between C21 and C20. **b**, As **a** but for midwinter (January-February).

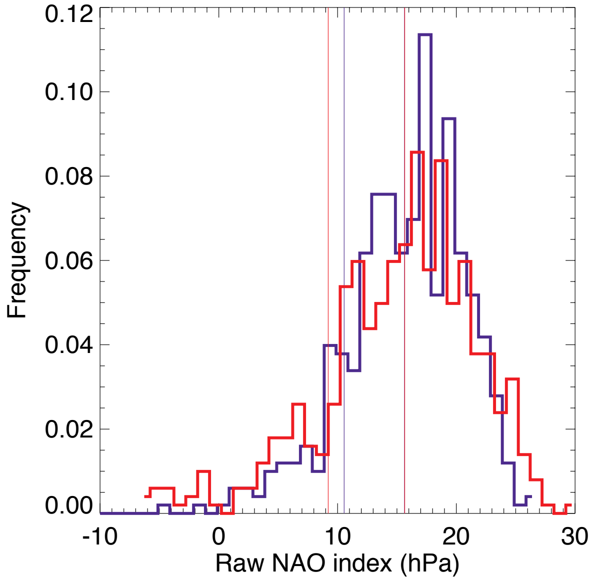

Supplementary Figure 3 | Climatological surface NAO response to Arctic sea ice loss. Probability distribution functions of the surface NAO index in the high ice (HI; blue) and low ice (LI; red) simulations. Vertical lines show the mean and one standard deviation below the mean. Note the means for LI and HI plot on top of each other.

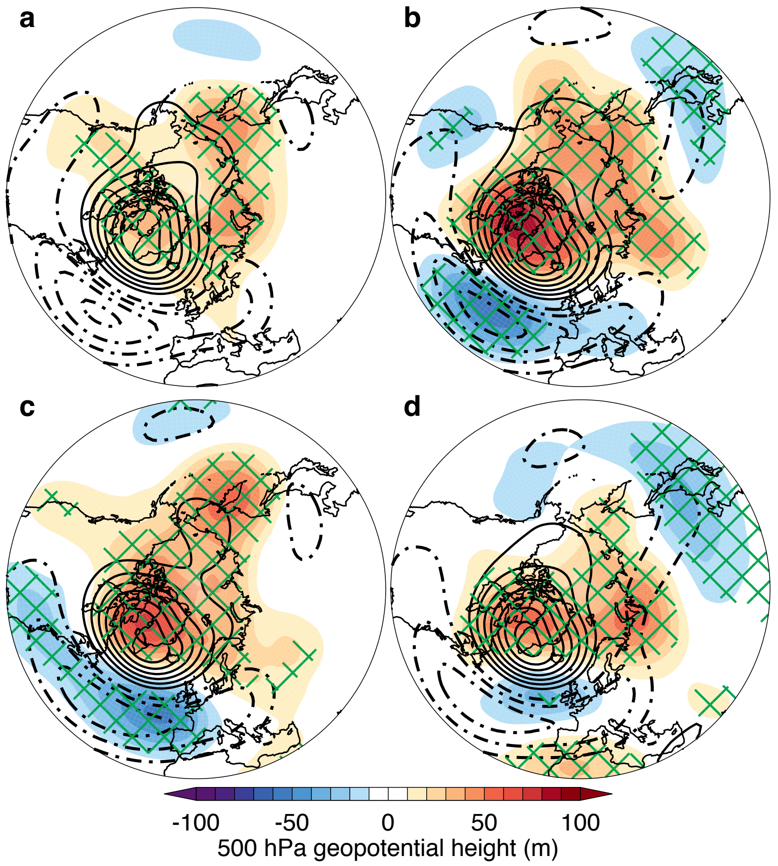

Supplementary Figure 4 | Monthly changes to NAO- events induced by Arctic sea ice loss. a, December 500 hPa geopotential height during NAO- events, differenced between the low ice (LI) and high ice (HI) simulations (shading; LI minus HI). Green hatching denotes differences that are statistically significant at the 95% (p = 0.05) confidence level. Black contours show the average geopotential height for NAO- events relative to climatology (average of both LI and HI; solid for positive; dashed for negative; drawn from -200 to 200 at intervals of 20 m, excluding zero). **b-d**, As **a** but for January, February and March, respectively.

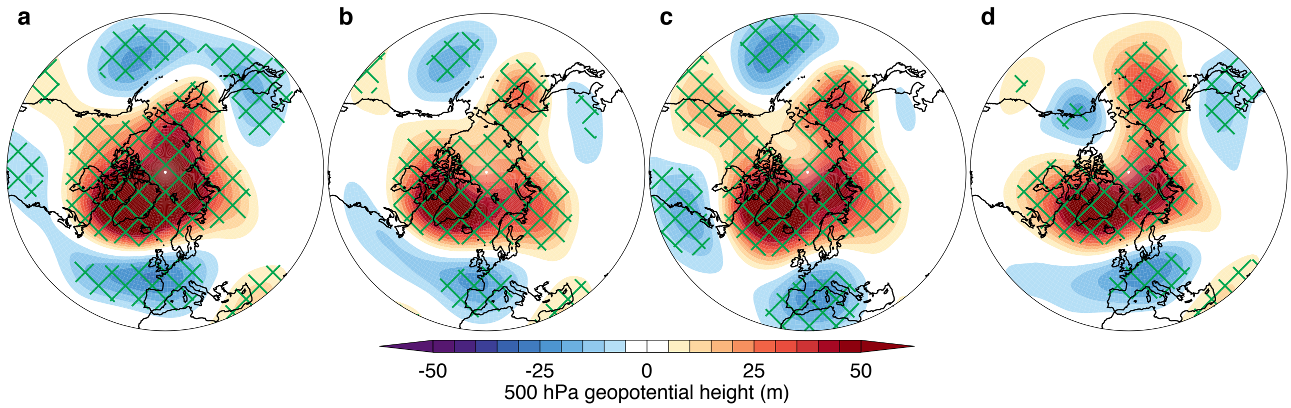

Supplementary Figure 5 | Climatological response to Arctic sea ice loss for different background states. a, Midwinter (January-February) 500 hPa geopotential height differenced between the low ice (LI) and high ice (HI) simulations (LI minus HI) for cases with a PDO- background SST state (see Methods). Green hatching denotes differences that are statistically significant at the 95% (p = 0.05) confidence level. **b-d**, As **a** but for PDO+, AMO- and AMO+ states.

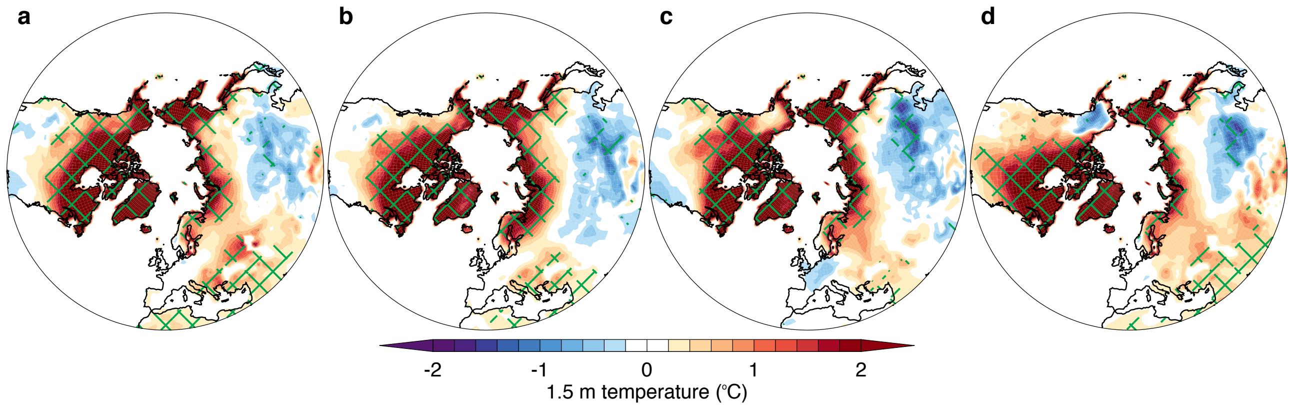

Supplementary Figure 6 | Climatological response to Arctic sea ice loss for different background states. a-d, As Supplementary Fig. 4, but for 1.5 m temperature.

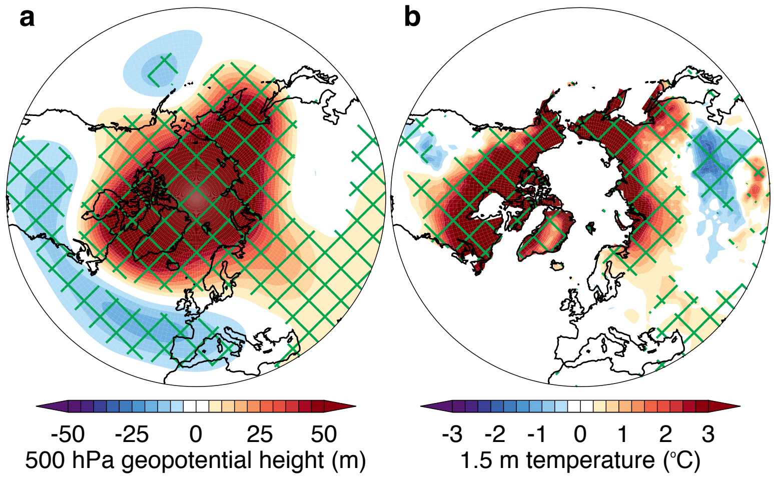

Supplementary Figure 7 | Climatological response to Arctic sea ice loss. a, Midwinter (January-February) 500 hPa geopotential height differenced between the 21st century (C21) and 20th century (C20) simulations (C21 minus C20). Green hatching denotes differences that are statistically significant at the 95% (p = 0.05) confidence level. **b**, As **a** but for 1.5 m temperature.

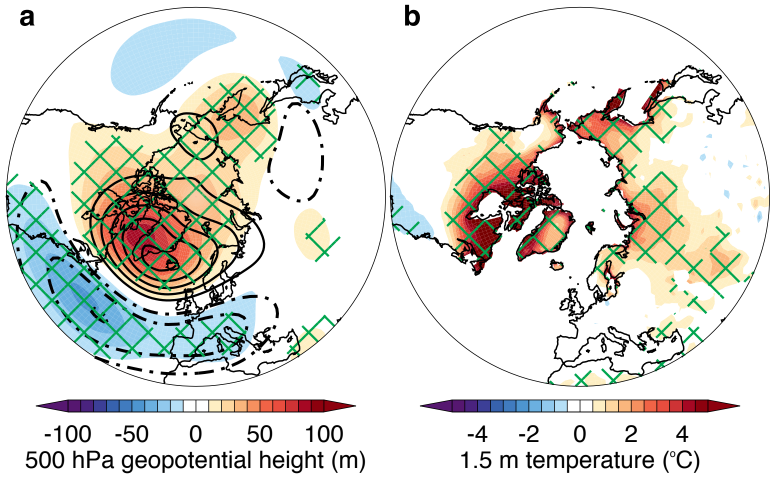

Supplementary Figure 8 | Changes to NAO- events induced by Arctic sea ice loss. **a**,**b**, As Fig. 2a and Fig. 4c, respectively, but selecting NAO- events in the high ice (HI) and low ice (LI) simulations using alternative NAO indices. The alternative NAO indices are defined based on the Principal Component time-series of the leading Empirical Orthogonal Function of sea level pressure (90 °W-40 °E 20-80 °N) rather than a latitudinal difference (see Methods).

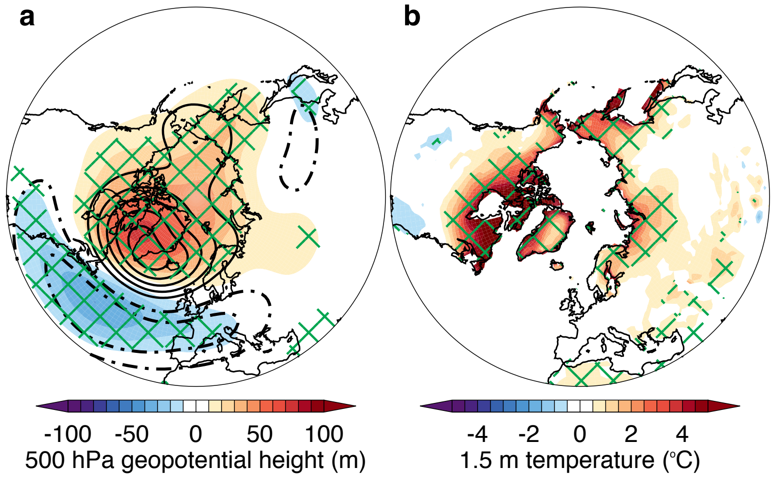

Supplementary Figure 9 | Changes to NAO- events induced by Arctic sea ice loss. **a**,**b**, As Fig. 2a and Fig. 4c, respectively, but selecting NAO- events in the low ice (LI) simulation using an alternative NAO index. The alternative NAO index is normalised relative to the high ice (HI) simulation rather than relative to LI (see Methods).
